# Peripartum cardiomyopathy and acute heart failure associated with prolonged tocolytic therapy in pregnancy: A case report

**DOI:** 10.1097/MD.0000000000027080

**Published:** 2021-08-27

**Authors:** Pei-Chen Li, Huai-Ren Chang, Sheng-Po Kao

**Affiliations:** a Department of Obstetrics and Gynecology, Hualien Tzu Chi Hospital, Buddhist Tzu Chi Medical Foundation, Hualien, Taiwan; b Division of Cardiology, Department of Internal Medicine, Hualien Tzu Chi Hospital, Buddhist Tzu Chi Medical Foundation, Hualien, Taiwan.

**Keywords:** acute heart failure, cervical insufficiency, peripartum cardiomyopathy, tocolysis

## Abstract

**Rationale:**

Peripartum cardiomyopathy (PPCM) is a rare and sometimes fatal systolic heart failure that affects women during late pregnancy or the early postpartum period. Heart failure symptoms can mimic the physiological changes of normal pregnancy, and the diagnosis is based on echocardiography.

**Patient concerns:**

A 38-year-old multiparous woman with a history of cervical incompetence underwent cervical cerclage and received tocolysis for 100 days.

**Diagnoses:**

She delivered vaginally at 37 weeks of gestation but developed postpartum decompensated acute heart failure with low left ventricular ejection fraction (LVEF: 34%) and was diagnosed with PPCM.

**Interventions:**

She received standard therapy for acute heart failure.

**Outcomes:**

The patient's pulmonary edema cleared, and she was fully ambulatory 6 days after admission. A follow-up echocardiogram 3 months later demonstrated recovery of LVEF to 66%.

**Lessons:**

Prolonged tocolysis may contribute to cardiomyopathy and should be used with caution. PPCM management requires standard treatments for acute heart failure with modifications for fetal safety.

## 1 Introduction

Peripartum cardiomyopathy (PPCM) is a rare, life-threatening form of dilated cardiomyopathy defined as systolic cardiac heart failure in the last month of pregnancy or the first 5 months after delivery.^[1]^ The incidence of PPCM has increased in the past decade and ranges from one in 1000 to 4000 live births in the United States.^[2]^ Risk factors include pre-eclampsia, advanced maternal age, multiparity, multifetal gestation, long-term tocolysis with beta-agonists, and African American ethnicity.^[3]^ Delayed diagnosis and treatment may result in irreversible cardiac dysfunction or mortality.^[2]^

Mid-trimester loss or premature birth can be caused by cervical insufficiency or a short and dilated cervix. Cerclage should be considered if the cervical length is ≤25 mm at less than 24 weeks.^[4]^

Tocolytics are frequent adjunctive therapy for patients who undergo cervical cerclage.^[5]^ However, adverse side effects can be caused by beta-2 adrenergic agonists, which are widely used tocolytic agents, including maternal pulmonary edema^[6]^ and vasodilatation, which leads to a compensatory increase in heart rate, stroke volume, cardiac output, and systolic blood pressure.^[7]^ Cardiovascular decompensation may occur after prolonged use of beta-2 adrenergic agonists. The direct effects of beta-2 adrenergic agonists on cardiovascular function have not been fully investigated.

We report the case of a woman who received tocolytic therapy for 3.5 months after cervical cerclage and presented with acute symptoms and signs of decompensated heart failure 3 days after delivery. The patient provided informed consent for the publication of the case.

## 2 Case report

A 38-year-old postpartum woman, gravida 3, para 2, ab 1, with no history of diabetes, hypertension, or cardiovascular disease was admitted to the hospital for acute dyspnea and leg edema.

The patient had a preterm delivery at 36 weeks of gestation in her first pregnancy 12 years ago, which did not require cervical cerclage or tocolysis. Her father had hypertension. She presented to the obstetric department at 21 weeks of gestation and underwent cerclage after her cervical length was found to be <19 mm. She was given tocolytic therapy with ritodrine, nifedipine, and diclofenac, which continued for 100 days until her delivery. The patient remained healthy until the last week of pregnancy when she developed mild dyspnea and increased leg swelling. She had a vaginal birth at 37 weeks of gestation.

**Figure 1:**
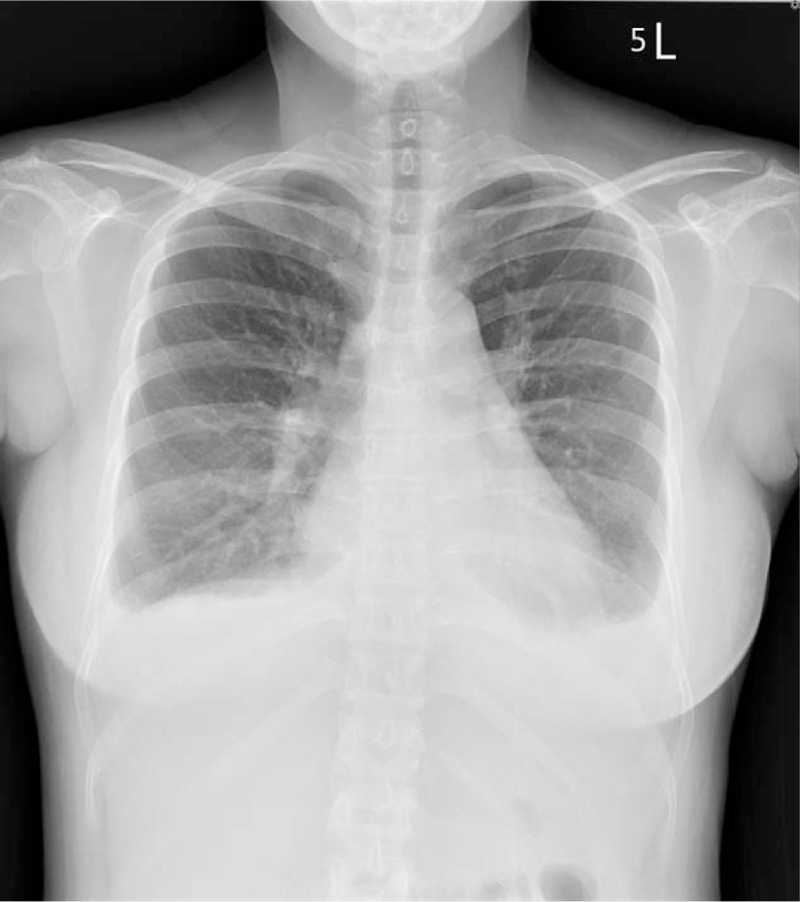
Chest x-ray showing cardiomegaly and pulmonary edema.

**Figure 2:**
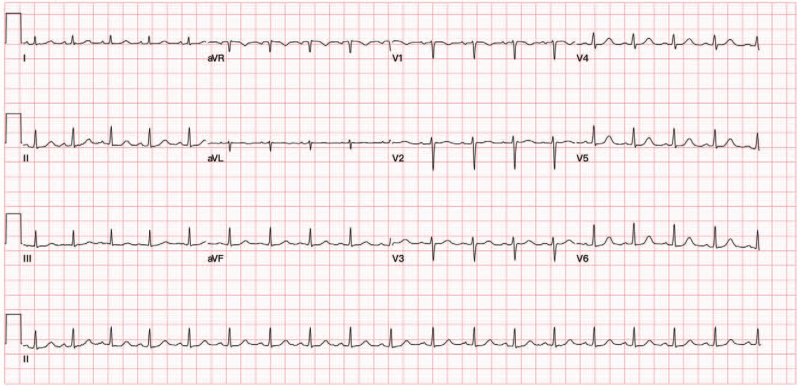
Twelve lead electrocardiograms showing sinus tachycardia at a rate of 111 beats per min with a QRS duration of 64 ms.

**Figure 3:**
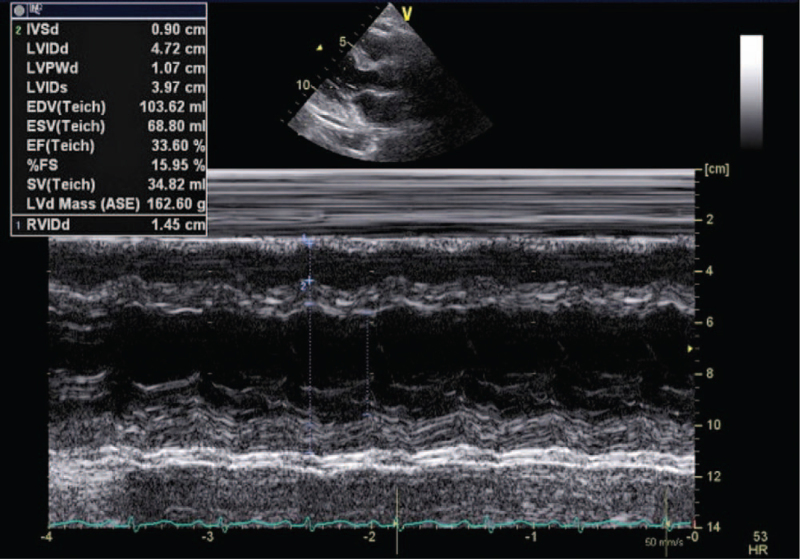
M-mode echocardiography demonstrating low left ventricular ejection fraction.

Three days after delivery, the patient presented to the emergency department with progressive dyspnea, orthopnea, and significant bilateral leg swelling. Her blood pressure was 161/110 mmHg, respiratory rate 30/min, and tachycardia 118/min. Laboratory data showed her troponin T level at 0.13 ug/l and N-terminal pro-brain natriuretic peptide level at 5,430 pg/ml. Chest imaging revealed pulmonary edema and bilateral pleural effusion (Fig. 1). A computed tomography pulmonary angiogram was negative for pulmonary emboli and electrocardiography revealed sinus tachycardia (Fig. 2). An echocardiogram showed generalized hypokinesia of left ventricular (LV) wall motion and a low ejection fraction of 34% (Fig. 3).

The patient was diagnosed with PPCM and given standard therapy for acute heart failure, including inotrope, vasodilators, ACE-inhibitors, beta-blockers, and diuretics; she improved clinically and the pulmonary edema cleared by the fifth day of hospitalization. The patient was fully ambulatory and was discharged 6 days after admission. A follow-up echocardiogram at 3 months and 12 months showed normalization of her ejection fraction to 64% and 80%, respectively. The patient showed complete recovery from PPCM and remained asymptomatic.

## 3 Discussion

We describe a multiparous female patient with cervical incompetence treated with a cervical cerclage and long-term tocolysis. Three days after delivery, she developed decompensated acute heart failure and was diagnosed with PPCM, which is a rare and sometimes fatal disease of unknown etiology. The 2010 Heart Failure Association of the European Society of Cardiology(ESC) Working Group defined PPCM as idiopathic cardiomyopathy with heart failure secondary to LV systolic dysfunction with a left ventricular ejection fraction (LVEF) of <45% during the last month of pregnancy or within 5 months following delivery. Their study finds no identifiable cause for heart failure but they speculate that it could be due to ventricular dilatation.^[8]^

The proposed pathogenesis of PPCM includes viral vasculitis,^[9]^ endothelial dysfunction,^[10]^ selenium deficiency,^[11]^ increased levels of prolactin,^[12]^ oxidative stresses from the vascular hormone soluble fms-like tyrosine kinase receptor 1,^[13]^ autoimmune response,^[14,15]^ or genetic predisposition.^[16,17]^ Previous studies have shown advanced age, black race, pre-eclampsia, hypertension, multiple gestations, anemia, and prolonged tocolysis to be risk factors for PPCM.^[18]^ Long-term use of tocolytic agents may contribute to cardiovascular dysfunction. A previous case series by Witlin et al reported PPCM in 2 patients out of 28 who had been administered tocolysis, including magnesium sulfate, ritodrine, terbutaline, and indomethacin.^[19]^ Lampert et al retrospectively reviewed 15 patients who had PPCM and found that 4 had undergone prolonged tocolytic therapy for 4 to 11 weeks, with a mean duration of treatment of 6.7 weeks.^[20]^

Cardiovascular events have been associated with ritodrine use in pregnancy, including chest pain, pulmonary edema, cardiac arrhythmias, and peripheral vasodilatation.^[21,22]^ Studies also found that ritodrine decreased cardiac baroreflex sensitivity, resulting in dysregulation of the vagal heart rate and increased blood pressure variability.^[7]^ Following a rise in heart rate, decompensated heart failure may occur because of reduced time for left atrial emptying, diastolic ventricular filling, and systolic ejection.^[6]^ On the other hand, β-adrenergic regulation also affects the balance between cardio-protection and cardiotoxicity.^[7,23]^ Cardiomyopathy may be triggered by beta-stimulation.

There is a high prevalence of pre-eclampsia in women with PPCM, and the 2 may share the same pathophysiology.^[24]^ Recent data suggest that antiangiogenic factor sFlt-1 from the placenta antagonizes circulating vascular endothelial growth factor and placental growth factor, leading to hypertension and endothelial dysfunction in pre-eclampsia. Higher serum soluble fms-like tyrosine kinase receptor 1 levels have also been found in women with PPCM, which correlates with poor myocardial recovery.^[13]^ However, our patient was treated with a calcium channel blocker to inhibit preterm labor, possibly concealing and delaying a pregnancy-induced hypertension diagnosis. Early detection of pre-eclampsia may help to enhance alertness and recognition of PPCM.

The management of PPCM requires a multidisciplinary team involving obstetricians, cardiologists, and anesthesiologists. The treatment goals are similar to non-pregnant acute heart failure with special consideration for fetal safety. Treatments for heart failure that are compatible with pregnancy status and breastfeeding include sodium restriction, loop diuretics, β blockers, hydralazine, nitrates, digoxin, and heparin. However, angiotensin-converting enzyme inhibitors and angiotensin receptor blockers are contraindicated during pregnancy due to fetotoxicity. According to ESC^[25]^ and American Heart Association^[26]^ guidelines, an urgent cesarean delivery should be considered in women presenting with acute heart failure and hemodynamic instability. In hemodynamically stable patients, vaginal delivery is preferable with epidural analgesia.

The reported mortality rate ranges from 2.0% to 12.6%^[25]^; however, recent evidence suggests that the LVEF recovers to a normal range within the first 3 to 6 months in 50% to 80% of women with PPCM.^[27]^ However, an LVEF of <30% at the time of diagnosis indicates lower recovery rates and increased adverse events.^[3]^ In addition, approximately 50% of recovered patients could experience recurrent PPCM and deterioration of LV function in subsequent pregnancies.^[28,29]^ Therefore, both the ESC and American Heart Association guidelines recommend contraception to PPCM patients without normalized LVEF.^[25,26]^

PPCM is a significant cause of maternal morbidity and mortality worldwide. The rising incidence of PPCM may be related to the higher prevalence of predisposing disorders such as advanced maternal age, hypertension, diabetes, and multifetal pregnancies, and possibly to increased recognition of the disease. A major challenge is distinguishing the physiologic peripartum changes in healthy women from the pathological symptoms of PPCM. Diagnosis of PPCM is difficult and requires close attention to heart failure signs and symptoms, especially in late pregnancy and the puerperium.

The present case was that of a multiparous woman of advanced maternal age, who did not have a history of hypertensive disease. Long-term intravenous tocolytic therapy not only results in fluid overload but also dysregulation of heart rate and blood pressure. Hemodynamic stress results in decompensated heart failure. As a result, tocolytic agents, especially β agonists, should be used with considerable caution in pregnant women with underlying cardiovascular disorders. The best prognosis can be achieved with early attention to cardiac failure symptoms in women who receive tocolysis and medical treatment.

## Author contributions

**Writing – original draft:** Pei-Chen Li.

**Writing – review & editing:** Huai-Ren Chang, Sheng-Po Kao.

## References

[1] Pearson GD Veille JC Rahimtoola S. Peripartum cardiomyopathy: National Heart, Lung, and Blood Institute and Office of Rare Diseases (National Institutes of Health) workshop recommendations and review. JAMA 2000;283:1183–8. 10703781 10.1001/jama.283.9.1183

[2] Gunderson EP Croen LA Chiang V Yoshida CK Walton D Go AS. Epidemiology of peripartum cardiomyopathy: incidence, predictors, and outcomes. Obstet Gynecol 2011;118:583–91. 21860287 10.1097/AOG.0b013e318229e6de

[3] Honigberg MC Givertz MM. Peripartum cardiomyopathy. BMJ 2019;364:k5287. 30700415 10.1136/bmj.k5287

[4] Brown R Gagnon R Delisle M-F. No. 373-Cervical insufficiency and cervical cerclage. J Obstet Gynaecol Can 2019;41:233–47. 30638557 10.1016/j.jogc.2018.08.009

[5] Smith J DeFranco EA. Tocolytics used as adjunctive therapy at the time of cerclage placement: a systematic review. J Perinatol 2015;35:561–5. 25905689 10.1038/jp.2015.38

[6] Lamont RF. The pathophysiology of pulmonary oedema with the use of beta-agonists. BJOG 2000;107:439–44. 10759259 10.1111/j.1471-0528.2000.tb13259.x

[7] Vesalainen RK Ekholm EM Jartti TT Tahvanainen KU Kaila TJ Erkkola RU. Effects of tocolytic treatment with ritodrine on cardiovascular autonomic regulation. Br J Obstet Gynaecol 1999;106:238–43. 10426643 10.1111/j.1471-0528.1999.tb08237.x

[8] Sliwa K Hilfiker-Kleiner D Petrie MC. Current state of knowledge on aetiology, diagnosis, management, and therapy of peripartum cardiomyopathy: a position statement from the Heart Failure Association of the European Society of Cardiology Working Group on peripartum cardiomyopathy. Eur J Heart Fail 2010;12:767–78. 20675664 10.1093/eurjhf/hfq120

[9] Fett JD. Viral infection as a possible trigger for the development of peripartum cardiomyopathy. Int J Gynaecol Obstet 2007;97:149–50. 17368646 10.1016/j.ijgo.2007.01.012

[10] Patten IS Rana S Shahul S. Cardiac angiogenic imbalance leads to peripartum cardiomyopathy. Nature 2012;485:333–8. 22596155 10.1038/nature11040 PMC3356917

[11] Fett JD Ansari AA Sundstrom JB Combs GF. Peripartum cardiomyopathy: a selenium disconnection and an autoimmune connection. Int J Cardiol 2002;86:311–6. 12419571 10.1016/s0167-5273(02)00359-5

[12] Hilfiker-Kleiner D Kaminski K Podewski E. A cathepsin D-cleaved 16 kDa form of prolactin mediates postpartum cardiomyopathy. Cell 2007;128:589–600. 17289576 10.1016/j.cell.2006.12.036

[13] Damp J Givertz MM Semigran M. Relaxin-2 and soluble Flt1 levels in peripartum cardiomyopathy: results of the multicenter IPAC study. JACC Heart Fail 2016;4:380–8. 26970832 10.1016/j.jchf.2016.01.004 PMC4851559

[14] Haghikia A Kaya Z Schwab J. Evidence of autoantibodies against cardiac troponin I and sarcomeric myosin in peripartum cardiomyopathy. Basic Res Cardiol 2015;110:60. 26519371 10.1007/s00395-015-0517-2

[15] Vilela EM Bettencourt-Silva R da Costa JT. Anti-cardiac troponin antibodies in clinical human disease: a systematic review. Ann Transl Med 2017;5:307. 28856147 10.21037/atm.2017.07.40 PMC5555984

[16] Canpolat U Çetin EH Yayla Ç Aras D. Familial occurrence of peripartum cardiomyopathy: genetic origin, unrecognized dilated cardiomyopathy or chance effect? J Cardiol Cases 2015;12:101–3. 30546567 10.1016/j.jccase.2015.05.002 PMC6281867

[17] Ware JS Seidman JG Arany Z. Shared genetic predisposition in peripartum and dilated cardiomyopathies. N Engl J Med 2016;374:2601–2. 10.1056/NEJMc1602671 27355546

[18] Lee S Cho GJ Park GU. Incidence, risk factors, and clinical characteristics of peripartum cardiomyopathy in South Korea. Circ Heart Fail 2018;11:e004134. 29626099 10.1161/CIRCHEARTFAILURE.117.004134

[19] Witlin AG Mabie WC Sibai BM. Peripartum cardiomyopathy: an ominous diagnosis. Am J Obstet Gynecol 1997;176:182–8. 9024111 10.1016/s0002-9378(97)80033-6

[20] Lampert MB Hibbard J Weinert L Briller J Lindheimer M Lang RM. Peripartum heart failure associated with prolonged tocolytic therapy. Am J Obstet Gynecol 1993;168:493–5. 8438916 10.1016/0002-9378(93)90479-3

[21] Neilson JP West HM Dowswell T. Betamimetics for inhibiting preterm labour. Cochrane Database Syst Rev 2014;CD004352. 24500892 10.1002/14651858.CD004352.pub3 PMC10603219

[22] Benedetti TJ. Maternal complications of parenteral beta-sympathomimetic therapy for premature labor. Am J Obstet Gynecol 1983;145:01–6. 10.1016/0002-9378(83)90331-9 6849333

[23] Fabry IG De Paepe P Kips JG Van Bortel LM. The influence of tocolytic drugs on cardiac function, large arteries, and resistance vessels. Eur J Clin Pharmacol 2011;67:573–80. 21494767 10.1007/s00228-011-1040-5

[24] Bello N Rendon ISH Arany Z. The relationship between pre-eclampsia and peripartum cardiomyopathy: a systematic review and meta-analysis. J Am Coll Cardiol 2013;62:1715–23. 24013055 10.1016/j.jacc.2013.08.717 PMC3931606

[25] Regitz-Zagrosek V Roos-Hesselink JW Bauersachs J. 2018 ESC guidelines for the management of cardiovascular diseases during pregnancy. Eur Heart J 2018;39:3165–241. 30165544 10.1093/eurheartj/ehy340

[26] Canobbio MM Warnes CA Aboulhosn J. Management of pregnancy in patients with complex congenital heart disease: a scientific statement for healthcare professionals from the American Heart Association. Circulation 2017;135:e50–87. 28082385 10.1161/CIR.0000000000000458

[27] McNamara DM Elkayam U Alharethi R. Clinical outcomes for peripartum cardiomyopathy in North America: results of the IPAC Study (investigations of pregnancy-associated cardiomyopathy). J Am Coll Cardiol 2015;66:905–14. 26293760 10.1016/j.jacc.2015.06.1309 PMC5645077

[28] Elkayam U. Risk of subsequent pregnancy in women with a history of peripartum cardiomyopathy. J Am Coll Cardiol 2014;64:1629–36. 25301468 10.1016/j.jacc.2014.07.961

[29] Hilfiker-Kleiner D Haghikia A Masuko D. Outcome of subsequent pregnancies in patients with a history of peripartum cardiomyopathy. Eur J Heart Fail 2017;19:1723–8. 28345302 10.1002/ejhf.808

